# OCT1 Is a Poor Prognostic Factor for Breast Cancer Patients and Promotes Cell Proliferation via Inducing NCAPH

**DOI:** 10.3390/ijms222111505

**Published:** 2021-10-25

**Authors:** Takuya Ogura, Kotaro Azuma, Junichiro Sato, Keiichi Kinowaki, Ken-Ichi Takayama, Toshihiko Takeiwa, Hidetaka Kawabata, Satoshi Inoue

**Affiliations:** 1 Department of Systems Aging Science and Medicine, Tokyo Metropolitan Institute of Gerontology, 35-2 Sakae-cho, Itabashi-ku, Tokyo 173-0015, Japan; ogutaku0120@gmail.com (T.O.); azumak@m.u-tokyo.ac.jp (K.A.); takayama@tmig.or.jp (K.-I.T.); ttakeiwa@tmig.or.jp (T.T.); 2 Department of Breast and Endocrine Surgery, Toranomon Hospital, 2-2-2 Toranomon, Minato-ku, Tokyo 105-8470, Japan; h-kawabata@toranomon.gr.jp; 3 Department of Systems BioMedicine, Tokyo Medical and Dental University, 1-5-45 Yushima, Bunkyo-ku, Tokyo 113-8510, Japan; 4 Department of Pathology, Toranomon Hospital, 2-2-2 Toranomon, Minato-ku, Tokyo 105-8470, Japan; j.n.sato@nifty.com (J.S.); kinowaki-hok@umin.ac.jp (K.K.); 5 Division of Systems Medicine and Gene Therapy, Saitama Medical University, 1397-1 Yamane, Hidaka-shi, Saitama 350-1241, Japan

**Keywords:** breast cancer, octamer transcription factor 1 (OCT1), non-structural maintenance of chromosomes condensin I complex subunit H (NCAPH), cell cycle, proliferation

## Abstract

Octamer transcription factor 1 (OCT1) is a transcriptional factor reported to be a poor prognostic factor in various cancers. However, the clinical value of OCT1 in breast cancer is not fully understood. In the present study, an immunohistochemical study of OCT1 protein was performed using estrogen receptor (ER)-positive breast cancer tissues from 108 patients. Positive OCT1 immunoreactivity (IR) was associated with the shorter disease-free survival (DFS) of patients (*p* = 0.019). Knockdown of OCT1 inhibited cell proliferation in MCF-7 breast cancer cells as well as its derivative long-term estrogen-deprived (LTED) cells. On the other hand, the overexpression of OCT1 promoted cell proliferation in MCF-7 cells. Using microarray analysis, we identified the non-structural maintenance of chromosomes condensin I complex subunit H (NCAPH) as a novel OCT1-taget gene in MCF-7 cells. Immunohistochemical analysis showed that *NCAPH* IR was significantly positively associated with OCT1 IR (*p* < 0.001) and that positive *NCAPH* IR was significantly related to the poor DFS rate of patients (*p* = 0.041). The knockdown of *NCAPH* inhibited cell proliferation in MCF-7 and LTED cells. These results demonstrate that OCT1 and its target gene *NCAPH* are poor prognostic factors and potential therapeutic targets for patients with ER-positive breast cancer.

## 1. Introduction

Breast cancer is one of the most frequent malignant diseases worldwide. Breast cancer is the most commonly diagnosed cancer and was the leading cause of cancer deaths in women in 2020 [1]. The number of patients is expected to increase further in the future [2]. Among them, about 80% of patients are estimated to have estrogen receptor (ER)-positive breast cancer [3]. In recent years, drug therapy for ER-positive breast cancer has evolved. In addition to conventional endocrine therapy and chemotherapy, molecular targeted therapies are under investigation, and some of them are available in clinical settings [4,5]. However, in a certain percentage of patients, the cancer develops endocrine resistance [6]. Therefore, the emergence of new drug therapies that will further prolong the survival of breast cancer patients is aspired to.

In this study, we investigated the clinical value of Octamer transcription factor 1 (OCT1) in ER-positive breast cancer. OCT1 is a transcriptional factor that is also known as POU domain class 2 transcription factor 1 (POU2F1). It binds specifically to the octamer consensus sequence (ATGCAAAT) in the promoter region of the DNA of the target genes and regulates their transcription [7]. Acyl-CoA synthetase long chain family member 3 (*ACSL3*) [8], disks large-associated protein 5 (*DLGAP5*) [9], anillin actin binding protein (*ANLN*) [10], and cyclin D1 (*CCND1*) [11], which are involved in cell proliferation, were reported as target genes. In addition to these genes, several genes related to various functions, such as cellular stress response, metabolic regulation, and cancer metastasis, have been reported as OCT1-target genes [12,13,14,15,16,17].

Oct1 has been shown to be a prognostic factor in various carcinomas. We have previously reported that the high expression of OCT1 is a prognostic factor in prostate cancer patients [18]. Additionally, in gastric cancer patients [19], colorectal cancer patients [20], and hepatocellular carcinoma patients [21], the high expression of OCT1 has been reported as a poor prognostic factor. As far as we know, the effect of OCT1 on the prognosis in breast cancer patients has not been reported upon so far. In the present study, we showed that the immunoreactivity of OCT1 is associated with the poor prognosis of ER-positive breast cancer patients. The function of OCT1 in ER-positive breast cancer cells were also studied.

## 2. Results

### 2.1. Positive OCT1 Immunoreactivity Was Associated with the Poor Prognosis of Breast Cancer Patients

**Figure 1:**
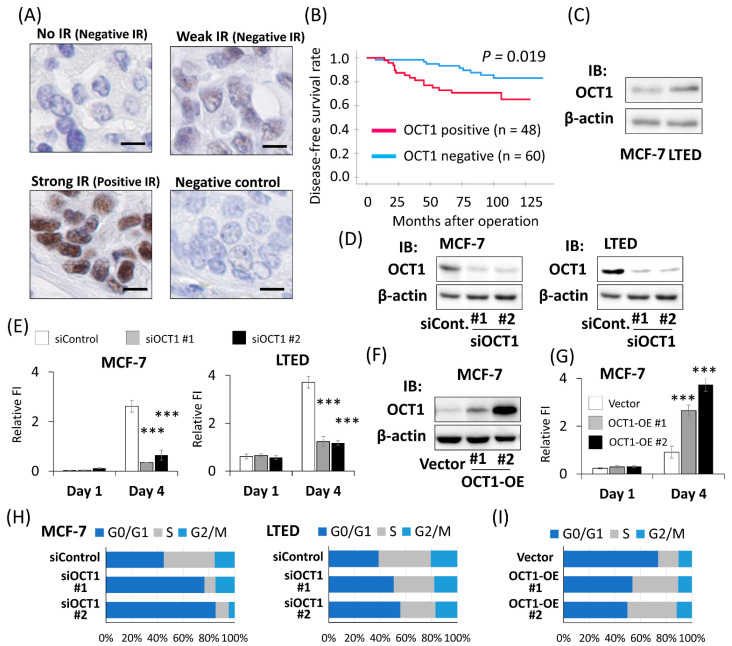
OCT1 was a poor prognostic factor in ER-positive breast cancer patients. (**A**) Representative micrographs of breast cancer tissues stained with OCT1 antibody. Strong immunoreactivity (IR) was defined as positive IR, whereas weak IR or no IR was defined as negative IR. A breast cancer tissue was applied to non-specific rabbit IgG antibody as a negative control. The scale bars represent 10 μm. (**B**) Disease-free survival of breast cancer patients with positive or negative OCT1 IR is shown by the Kaplan-Meier method. *p*-value was determined by the log-rank test. The red line represents cases with positive OCT1 IR (*n* = 48), and the blue line represents negative OCT1 IR (*n* = 60). (**C**) Western blot analysis for OCT1 expression in MCF-7 cells and LTED cells. β-actin protein was blotted as a loading control. IB, immunoblot. (**D**) Western blot analysis for OCT1 expression in MCF-7 cells and LTED cells treated with two kinds of siRNAs for OCT1 (siOCT1 #1 or #2) or siControl (siCont.). β-actin protein was blotted as a loading control. (**E**) DNA content of MCF-7 and LTED cells on indicated days after transfection of indicated siRNAs analyzed by Hoechst 33342 staining. Relative fluorescence intensity (FI) was shown as mean and SEM (*n* = 4). *** *p* < 0.001 compared to cells treated with siControl. (**F**) Western blot analysis for OCT1 expression in two clones of MCF-7 cells stably expressing OCT1 (OCT1-OE #1 and #2) and an MCF-7 clone transfected with empty vector (Vector). β-actin protein was blotted as a loading control. (**G**) DNA content of the OCT1-OE clones (#1 and #2) and the vector clone (Vector) on indicated days after seeding was analyzed by Hoechst 33342 staining. Relative fluorescence intensity (FI) was shown as mean and SEM (*n* = 4). *** *p* < 0.001 compared to the vector clone. (**H**) Proportions of cell populations in G0/G1, S, and G2/M phases of the cell cycle in MCF-7 and LTED cells transfected with indicated siRNAs. The results of flow cytometric analysis shown in Figure S4B were quantified. (**I**) Proportions of cell populations in G0/G1, S, and G2/M phases of the cell cycle in the OCT1-OE clones (#1 and #2) and the vector clone (Vector). The results of flow cytometric analysis shown in Figure S4C were quantified.

To explore the clinical value of OCT1 expression in ER-positive breast cancer, immunohistochemical analysis was performed with 108 female patients with ER-positive breast cancer who had undergone the surgical resection of primary tumors. Prior to immunostaining, the specific reactivity of the antibody against OCT1 was confirmed by Western blotting using cell lysate derived from HEK293 cells and MCF-7 cells overexpressing human OCT1 (Figure S1). OCT1 staining was observed in the nucleus (Figure 1A). No significant relationship was found between OCT1 IR and any clinicopathological parameters that were analyzed (Table 1). In terms of the relationship between OCT1 IR and clinical prognosis, positive OCT1 IR was significantly associated with a shorter DFS (*p* = 0.019) (Figure 1B). Univariate analysis using the Cox proportional hazard model demonstrated that OCT1 status, invasive tumor size, lymph node status, and nuclear grade were significant prognostic factor for DFS. Multivariate analysis of these factors showed that OCT1 status was the only independent prognostic factor (Table 2). The present results of OCT1 IR were in line with the analysis of *OCT1* mRNA expression using public databases. High expression of *OCT1* was significantly associated with poor prognosis in KM Plotter [22] (Figure S2A). According to the expression analysis using Oncomine [23], the *OCT1* mRNA expression level was significantly increased in invasive ductal breast cancer tissues compared to in the normal breast tissues (Figure S2B). We compared endogenous OCT1 expression in MCF-7 cells, which are ER-positive breast cancer cells, and their derivative LTED cells, a model of endocrine therapy-resistant breast cancer. Both mRNA and the protein expression levels of OCT1 were higher in LTED cells compared to those in MCF-7 cells Figure S3A and Figure 1C).

### 2.2. OCT1 Promoted the Cell Proliferation of Breast Cancer Cells

We investigated the biological function of OCT1 in ER-positive breast cancer cells. We performed the knockdown of OCT1 using siRNAs (siOCT1 #1, siOCT1 #2) in MCF-7 and LTED cells (Figure S3B and Figure 1D). The fluorescence intensity of Hoechst 33342 on the fourth day after siRNA transfection was decreased by silencing OCT1 in both MCF-7 and LTED cells (Figure 1E). Then, the effect of OCT1 knockdown on cell viability was analyzed by means of 3-(4,5-dimethylthiazol-2-yl)-5-(3-carboxymethoxyphenyl)-2-(4-sulfophenyl)-2H-tetrazolium (MTS) assay. The absorbance of 490 nm on the fourth day after siRNA transfection was suppressed by OCT1 knockdown in both MCF-7 and LTED cells (Figure S4A). Then, we established two clones of MCF-7 cells stably expressing OCT1 and an MCF-7 clone transfected with an empty vector (Figure S3C and Figure 1F). The fluorescence intensity of Hoechst 33342 on the fourth day after seeding was increased by OCT1 overexpression (Figure 1G). Next, we investigated the effect of OCT1 on the cell cycle of breast cancer cells using flow cytometry. The percentage of cells in the G0/G1 phase increased and that of cells in the S phase decreased in the cells treated by siOCT1 (Figure 1H and Figure S4B). Then, we examined the effect of OCT1 overexpression on the cell cycle. The percentage of cells in the G0/G1 phase decreased and that in the S phase increased in the OCT1 overexpressing cells (Figure 1I and Figure S4C).

### 2.3. Identification of OCT1-Induced Genes

**Figure 2:**
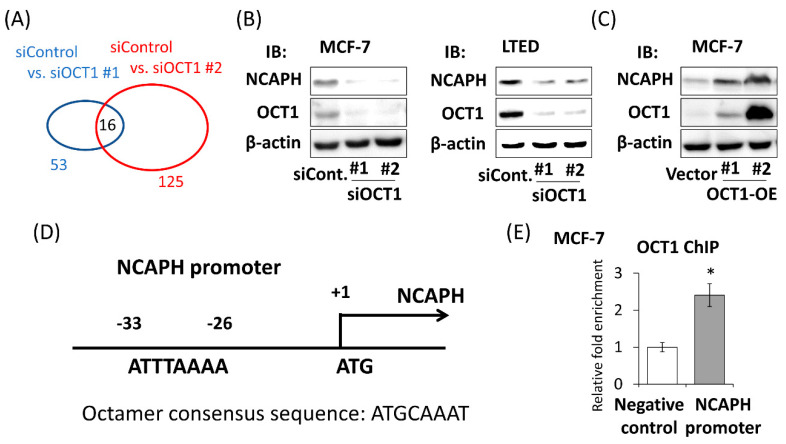
*NCAPH* was one of the OCT1-regulated genes in MCF-7 cells. (**A**) The results of microarray analyses were summarized. Genes downregulated with a fold change ≥8 by silencing OCT1 with siOCT1 #1 (blue oval) or with siOCT1 #2 (red oval) compared to treatment with siControl are shown. The digits indicate the number of genes. Sixteen genes were downregulated in common. (**B**) Western blot analysis for *NCAPH* and OCT1 expressions in MCF-7 cells and LTED cells treated with two kinds of siRNAs for OCT1 (siOCT1 #1 or #2) or siControl (siCont.). β-actin protein was blotted as a loading control. IB, immunoblot. (**C**) Western blot analysis for *NCAPH* and OCT1 expressions in two clones of MCF-7 cells stably expressing OCT1 (OCT1-OE #1 and #2) and an MCF-7 clone transfected with empty vector (Vector). β-actin protein was blotted as a loading control. (**D**) Schema of *NCAPH* promoter region. A putative octamer consensus sequence (ATTTAAAA) exists at 26 base pairs upstream from the translation initiation site (ATG) of *NCAPH* gene. (**E**) Association of OCT1 in the promoter region of *NCAPH* in MCF-7 cells. ChIP assay for OCT1 or normal rabbit IgG was performed. The fold enrichments relative to IgG in *NCAPH* promoter and another locus in *NCAPH* (negative control) were measured by performing quantitative PCR (qPCR). Relative fold enrichment was shown as mean and SEM (*n* = 3). * *p* < 0.05.

To further investigate the function of OCT1 in breast cancer cells, microarray analysis was performed using MCF-7 cells treated with siControl, siOCT1 #1, or siOCT1 #2. We compared the gene expression of siControl-treated cells with siOCT1 #1-treated cells and detected 53 genes as genes that are downregulated with an eight-fold change or more. We compared the gene expression of siControl-treated cells with siOCT1 #2-treated cells and detected 125 genes as genes that are downregulated with an eight-fold change or more. Among these genes, 16 genes were extracted in common (Figure 2A and Table S1). A total of 5 of 16 genes, namely ribonucleotide reductase regulatory subunit M2 (*RRM2*), cell division cycle associated 3 (*CDCA3*), non-SMC condensin I complex subunit H (*NCAPH*), centrosomal protein 55 (*CEP55*), and kinesin family member 20A (*KIF20A*), were associated with a poor prognosis of ER-positive breast cancer in a public dataset using KM Plotter (Figure S5A–E). These are novel candidates of OCT1-induced genes that potentially mediate the tumor promoting effect of OCT1. Among these genes, we focused on NCAPH, of which no functional analysis in breast cancer has been reported so far to the best of our knowledge. *NCAPH* is one of the subunits of condensin I, which is involved in the condensation and stabilization of chromosomes during mitosis [24,25]. To confirm the result of the microarray analysis, we evaluated the change of the *NCAPH* expression level when the expression of OCT1 was manipulated. Both mRNA and protein expression levels were suppressed by silencing OCT1 in MCF-7 and LTED cells (Figure S7A and Figure 2B). On the other hand, the mRNA and protein expression levels of *NCAPH* were elevated in the OCT1 overexpressing MCF-7 cells in proportion to its expression level (Figure S7B and Figure 2C).

In breast cancer cell lines, it has been reported that OCT1 binds to the promoter region of *ESR1* encoding ERα and induces its transcription [26,27]. To determine whether the regulation of *NCAPH* is mediated by ERα, MCF-7 cells were treated with estradiol (E2). The expression of *NCAPH* was not affected by E2 treatment in the condition where *GREB1*, a typical estrogen-responsive gene, was significantly affected (Figure S7C,D). To investigate the possibility that OCT1 directly regulates the transcription of *NCAPH*, we searched the *NCAPH* promoter region to determine whether octamer consensus sequences existed in the region. We found a putative octamer consensus sequence at 26 base pairs upstream from the translation initiation site (Figure 2D). Then, we performed a chromatin immunoprecipitation (ChIP) assay to investigate whether OCT1 is associated with the *NCAPH* promoter region. In MCF-7 cells, the fold enrichment relative to IgG control in the *NCAPH* promoter was significantly higher compared to that in the irrelevant region in the first exon of the *NCAPH* gene (negative control) (Figure 2E).

### 2.4. Positive NCAPH Immunoreactivity Was Associated with the Poor Prognosis of Breast Cancer Patients

**Figure 3:**
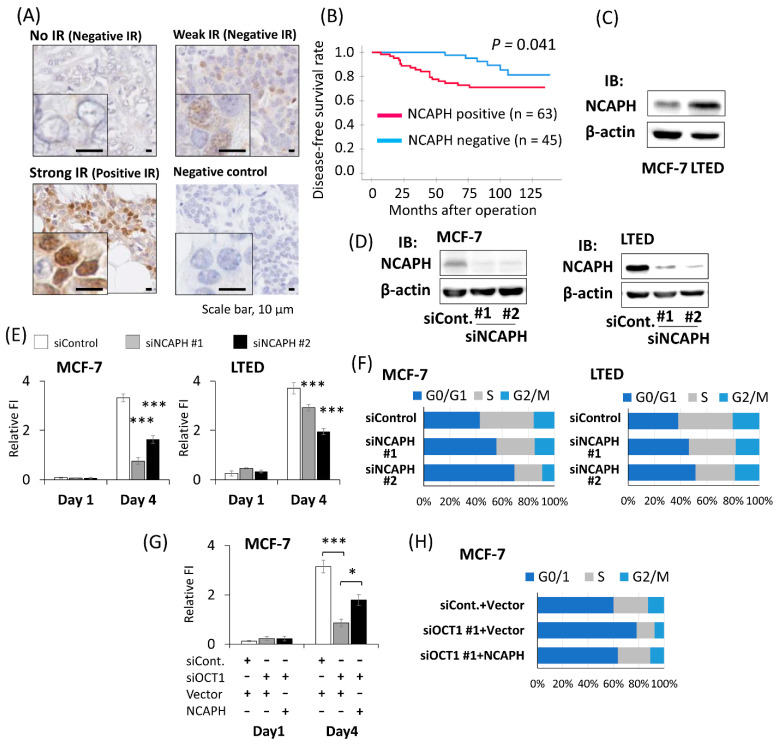
*NCAPH* was a poor prognostic factor in ER-positive breast cancer patients. (**A**) Representative micrographs of breast cancer tissues stained with *NCAPH* antibody. Strong immunoreactivity (IR) was defined as positive IR, whereas weak IR or no IR was defined as negative IR. One breast cancer tissue was applied with non-specific rabbit IgG antibody as a negative control. The scale bars represent 10 μm. (**B**) Disease-free survival of breast cancer patients with positive or negative *NCAPH* IR is shown by the Kaplan-Meier method. *p*-value was determined by the log-rank test. The red line represents cases with positive OCT1 IR (*n* = 63), and the blue line represents negative *NCAPH* IR (*n* = 45). (**C**) Western blot analysis for *NCAPH* expression in MCF-7 cells and LTED cells. β-actin protein was blotted as a loading control. IB, immunoblot. (**D**) Western blot analysis for *NCAPH* expression in MCF-7 cells and LTED cells treated with two kinds of siRNAs for *NCAPH* (si*NCAPH* #1 or #2) or siControl (siCont.). β-actin protein was blotted as a loading control. (**E**) DNA content of MCF-7 and LTED cells on indicated days after transfection of indicated siRNAs analyzed by Hoechst 33342 staining. Relative fluorescence intensity (FI) was shown as mean and SEM (*n* = 4). *** *p* < 0.001 compared to cells treated with siControl. (**F**) Proportions of cell populations in G0/G1, S and G2/M phase of cell cycle in MCF-7 and LTED cells transfected with indicated siRNAs. The results of flow cytometric analysis shown in Figure S8 were quantified. (**G**) DNA content of MCF-7 cells treated with indicated siRNAs and expression vectors on indicated days after transfection of siRNAs was analyzed by Hoechst 33342 staining. On the day 0, transfection with siRNAs (siControl or siOCT1 #1) was performed. On the first day (Day1), transfection with expression vector encoding *NCAPH* (NCAPH) or empty vector (Vector) was performed. Relative fluorescence intensity (FI) was shown as mean and SEM (*n* = 4). * *p* < 0.05, *** *p* < 0.001. (**H**) Proportions of cell populations in G0/G1, S and G2/M phase of cell cycle in MCF-7 cells transfected with indicated siRNAs and expression vectors. The results of the flow cytometric analysis shown in Figure S9 were quantified.

To explore the clinical value of *NCAPH* expression in ER-positive breast cancer, immunohistochemical analysis was performed with the same 108 female breast cancer patients as in the analysis of OCT1. Prior to immunostaining, the specific reactivity of the antibody against *NCAPH* was confirmed by Western blotting using cell lysate derived from HEK293 cells and MCF-7 cells overexpressing human *NCAPH* (Figure S6). *NCAPH* staining was observed in both the nucleus and in the cytoplasm. In some cases, the nuclei were prominently stained (Figure 3A). *NCAPH* IR was significantly positively associated with OCT1 status (*p* < 0.001) (Table 3). In terms of relationship between *NCAPH* IR and clinical prognosis, positive *NCAPH* IR was significantly associated with a shorter DFS (*p* = 0.041) (Figure 3B). Univariate analysis using the Cox proportional hazard model demonstrated that *NCAPH* status, invasive tumor size, lymph node status, and nuclear grade were significant prognostic factors for DFS. Multivariate analysis of these factors showed that *NCAPH* status and invasive tumor size were independent prognostic factors (Table 4). These immunohistochemical results were in line with the prognostic analysis conducted using KM plotter (Figure S5C). According to the expression analysis using Oncomine, the *NCAPH* mRNA expression level was significantly increased in the invasive ductal breast cancer tissues compared to in normal breast tissue (Figure S7E). Then, we compared endogenous *NCAPH* expression in the human breast cancer cell line MCF-7 and LTED cells. Both the mRNA and protein expression levels in LTED cells were higher than those in MCF-7 cells (Figure S7F and Figure 3C).

### 2.5. Knockdown of NCAPH Suppressed the Proliferation of Breast Cancer Cells

To investigate the biological functions of *NCAPH* in breast cancer cells, we performed the knockdown of *NCAPH* using siRNAs (si*NCAPH* #1, si*NCAPH* #2) in MCF-7 cells and LTED cells. We confirmed that both the mRNA and protein expression levels of *NCAPH* were suppressed by siRNAs (Figure S7G and Figure 3D). The fluorescence intensity of Hoechst 33342 on the fourth day after siRNA transfection was decreased by silencing the *NCAPH* in both the MCF-7 and LTED cells (Figure 3E). When the cell viability was assessed using an MTS assay, an absorbance of 490 nm on the fourth day after siRNA transfection was suppressed by *NCAPH* knockdown in both the MCF-7 and LTED cells (Figure S8A). Then, we examined the effect of the knockdown of *NCAPH* on the cell cycle using flow cytometry. The percentage of cells in the G0/G1 phase increased, and the percentage of cells in the S phase decreased in the cells treated with si*NCAPH* (Figure 3F and Figure S8B).

Then, we overexpressed *NCAPH* in the MCF-7 cells where OCT1 was knocked down. The exogenous *NCAPH* partially rescued the suppressed fluorescence intensity of Hoechst 33342 on the fourth day after siOCT1 transfection (Figure 3G). When the cell cycle was analyzed via flow cytometry, *NCAPH* overexpression reversed the increased percentage of the cells in the G0/G1 phase and the decreased percentage of the cells in the S phase by knocking down OCT1 (Figure S9 and Figure 3H).

## 3. Discussion

In the present study, we demonstrated that positive OCT1 IR was associated with worse prognosis in ER-positive breast cancer patients. We also demonstrated that positive OCT1 IR was an independent predictive factor among other clinicopathological prognostic factors. As far as we know, this is the first report on the clinical value of OCT1 immunohistochemistry for breast cancer. We observed increased OCT1 expression in LTED cells. Considering that LTED cells are assumed as a refractory model for endocrine therapy with aromatase inhibitor, our clinical results suggest the possible involvement of OCT1 in the endocrine resistance that might occur in breast cancer.

As a transcription factor, OCT1, is reported to regulate various genes [8,9,10,11,12,13,14,15,16,17]. Here, we added novel target gene candidates including *RRM2*, *CDCA3*, *NCAPH*, *CEP55*, and *KIF20A*. Through an analysis using a public database, we showed that the high expression of these genes is associated with the poor prognosis of ER-positive breast cancer patients. It may be assumed that the induction of these genes mediates cancer promotive effects of OCT1 in breast cancer cells, which is further supported by previous reports on the functions of these genes [28,29,30,31], with the exception of NCAPH. Since information on the roles of *NCAPH* in breast cancer was limited, we further analyzed the clinical value of NCAPH. With immunohistochemical analysis, we demonstrated that positive *NCAPH* IR was associated with the poor prognosis of ER-positive breast cancer patients. We also revealed that positive *NCAPH* IR was an independent predictive factor among other clinicopathological prognostic factors. In this analysis, we observed a significant positive correlation between OCT1 IR and *NCAPH* IR, which was in line with *NCAPH* being an OCT1-induced gene.

We demonstrated that the knockdown of *NCAPH* suppressed the proliferation of breast cancer cells. The growth promoting effect of *NCAPH* was also reported in several other cancer cells [32,33,34,35,36]. *NCAPH* is one of the subunits of condensin I, which is responsible for the condensation and stabilization of chromosomes in the M phase of the cell cycle [24,25]. In fact, in colorectal cancer cells, the knockdown of *NCAPH* induced cell cycle arrest at the G2/M phase [33]. In contrast, our data suggest that *NCAPH* promotes the transition from the G1 to the S phase in breast cancer cells. This might indicate that *NCAPH* has novel functions in the nucleus during the interphase other than regulating mitosis in the M phase. In recent years, it has been reported that condensin I may be involved in gene regulation [37,38], which suggests that an unknown function of condensin I exists during interphase. Meanwhile, NCAPG, another subunit of condensin I, has been reported to regulate the G1 phase in gastric cancer [39,40], which may further support the novel function of condensin I in the interphase. We showed that OCT1 promotes the proliferation of breast cancer cells by inducing a cell cycle transition from the G1 phase to the S phase, which was a similar target of action to NCAPH. These observations could be consistent with the hypothesis that the growth promoting effect of OCT1 is mediated by its transcriptional induction of NCAPH.

Our data showing a higher expression level of *NCAPH* in LTED cells compared to MCF-7 cells may suggest that *NCAPH* is related to endocrine resistance in breast cancer. It was reported that OCT1 binds to the promoter region of *ESR1* and up-regulates ERα in breast cancer cells [26,27], which implies that some of the OCT1 induced genes may be dependent on ERα expression. In the case of NCAPH, the induction of *NCAPH* by OCT1 would be independent from ERα expression since we showed *NCAPH* is not an estrogen responsive gene. Our results from the ChIP assay showed OCT1 binding in the *NCAPH* promoter region, which suggests that the expression of *NCAPH* could be directly regulated by OCT1. We could speculate that the high expression of *NCAPH* predicts poor prognosis for breast cancer patients because the expression of *NCAPH* was not suppressed by endocrine therapy. In the present study, we showed that the silencing OCT1 or *NCAPH* suppressed the proliferation of MCF-7 and LTED cells, which suggests that OCT1 and *NCAPH* could be potential targets for breast cancer therapy. Our data will provide clue for developing an effective strategy for patients with ER-positive breast cancer.

## 4. Materials and Methods

### 4.1. Collection of Human Tissue Samples and Clinical Data

Tissue samples of ER-positive invasive breast cancer were obtained form 108 Japanese female breast cancer patients who had undergone surgical resection from 2006 to 2013 at Toranomon Hospital, Tokyo, Japan. No patients received preoperative endocrine therapy, chemotherapy, or molecular target therapy. All patients received postoperative standard adjuvant therapy according to the clinical practice guidelines of the National Comprehensive Cancer Network (USA) [41]. The staging of all of the breast cancer cases was re-evaluated in 2018 according to the “TNM classification of Malignant Tumours” (8th Edition) [42]. The clinical outcome was evaluated by disease-free survival (DFS). DFS was determined as the time span from the date of surgery to the first recurrence or last follow-up. The mean follow-up duration was 99 months (range 19–139 months). Signed informed consent was obtained from all patients. This study was approved by the Ethics Committees of Toranomon Hospital (approval number: 845/1327, approval dates: 3 April 2017/22 June 2018) and the Tokyo Metropolitan Institute of Gerontology (approval number: J48, approval date: 23 January 2019). All procedures performed in this study were in accordance with the Declaration of Helsinki.

### 4.2. Antibodies

Rabbit monoclonal antibodies for estrogen receptor alpha (ERα) (clone: SP1), progesterone receptor (PgR) (clone: 1E2), and human epidermal growth factor receptor 2 (HER2) (clone: 4B5) were purchased from Roche (Basel, Switzerland). Mouse monoclonal anti-β-actin antibody (A2228) was obtained from Sigma-Aldrich (St. Louis, MO, USA). Rabbit polyclonal anti-OCT1 antibody (ab15112) was purchased from Abcam (Cambridge, MA, USA). Rabbit polyclonal anti-*NCAPH* antibody (11515-1-AP) was obtained from Proteintech (Chicago, IL, USA). Rabbit polyclonal anti-IgG antibody (#2729) was purchased from Cell Signaling Technology (Danvers, MA, USA)

### 4.3. Immunohistochemical Analysis

Immunohistochemical analysis of OCT1 and *NCAPH* expression was performed using an EnVision+ visualization kit (Dako, Carpinteria, CA, USA). The tissue sections (4 μm) were deparaffinized, rehydrated with a graded ethanol series, and rinsed in pure water. For antigen retrieval, the sections were pretreated by being heated in a water bath at 95 °C for 30 min in 20 mM Envision FLEX Target Retrieval Solution Low pH buffer (Dako). After blocking the endogenous peroxidase with 3% H_2_O_2_, the primary antibodies against OCT1 (1:1600 dilution) or *NCAPH* (1:200 dilution) were applied to the sections, and they were incubated overnight at 4 °C. The sections were rinsed in EnVision FLEX Wash Buffer (Dako) and were incubated with EnVision + Dual Link System-HRP (Dako) for 30 min at room temperature. The antigen-antibody complex was visualized using the Dako Liquid DAB + Substrate Chromogen System. For negative controls, normal rabbit IgG was used instead of primary antibodies. Immunostained slides were evaluated for intensity and proportion. Staining intensity was classified as none, weak, and strong. Staining proportion was measured by the percentage of stained tumor cells. The immunoreactivity (IR) of OCT1 was defined as “positive” when the nuclei of over 1/3 of the tumor cells were stained as strong [43]. The IR of *NCAPH* was defined as “positive” when the nuclei of more than 1% of the tumor cells were stained as strong [44]. Two pathologists (T.O. and K.K.) evaluated the slides, and in case of disagreement between the two pathologists, a third pathologist (J.S.) evaluated the IR.

Immunostaining of ER, PgR, and HER2 was performed automatically using VENTANA BenchMark GX (Roche). ER and PgR status were judged as positive when nuclear staining of more than 1% of the tumor cells was observed according to the guidelines of the American Society of Clinical Oncology/College of American Pathologists (ASCO/CAP) [45]. HER2 status was judged based on the updated guidelines from ASCO/CAP [46].

### 4.4. Cell Culture

Human breast cancer cell line MCF-7 was obtained from American Type Culture Collection (Manassas, VA, USA). The HEK293 cell line was obtained from Clontech (Palo Alto, CA, USA). Short tandem repeat-based authentication of the cell line was verified by BEX Co., Ltd. (Tokyo, Japan). Long-term estrogen-deprived (LTED) cells were established through the long-term (>4 months) culturing MCF-7 cells without estrogen [47]. MCF-7 cells and HEK293 cells were cultured in Dulbecco’s modified Eagle’s medium (DMEM) supplemented with 10% fetal bovine serum (FBS) and 1% penicillin-streptomycin (Nacalai Tesque, Kyoto, Japan) at 37 °C with 5% CO_2_. LTED cells and estrogen-treated cells were cultured in phenol red-free DMEM containing 5% charcoal-stripped FBS and 1% penicillin-streptomycin at 37 °C with 5% CO_2_. DMEM was purchased from Nacalai Tesque. Estradiol (E2) was purchased from Sigma-Aldrich.

### 4.5. Quantitative Reverse Transcription Polymerase Chain Reaction (qRT-PCR) Analysis

Total RNA extraction was performed using Sepasol-RNA I super G (Nacalai Tesque) according to the manufacturer’s protocols and was followed by cDNA synthesis using PrimeScript (Takara, Kyoto, Japan). The cDNA was subjected to qRT-PCR using Applied Biosystems StepOnePlus (Thermo Fisher Scientific, Waltham, MA, USA) based on the detection of SYBR Green fluorescence (Kapa Biosystems, Wilmington, MA, USA). mRNA expression levels were normalized with *GAPDH* using the 2-^ΔΔCt^ method [48]. The sequences of the primers were as follows:

*GAPDH* forward: 5′-TCTAGTAAAGTGGATATTGTTG-3′;

*GAPDH* reverse: 5′-GATGGTGATGGGATTTCC-3′;

*OCT1* forward: 5′-GATGGCACCCTCACAGTTTG-3′;

*OCT1* reverse: 5′-GCTCATTAGAGCTGGGCTGA-3′;

*NCAPH* forward: 5′-CTGATGGAAGTGCTACTGAAATGG-3′;

*NCAPH* reverse: 5′-TCTGAAACATGGGATCAATCTCAC-3′;

*GREB1* forward: 5′-GCTGTCGGAGTTTATTGAATCCAC-3′;

*GREB1* reverse: 5′-GCACGAGAACAAAGGTCCTG-3′.

### 4.6. Western Blot Analysis

Whole-cell lysates were prepared using lysis buffer containing 50 mM 4-(2-hydoroxyethyl)-1-piperazinly ethane-2-suffonic acid (HEPES), 150 mM NaCl, 10% glycerol, 1% Triton X-100, 1.5 mM MgCl_2_, and 1 mM ethylene glycol-bis(β-aminoethyl ether)-N,N,N’,N’-tetraacetic acid (EGTA). A proteinase inhibitor cocktail (Nacalai Tesque) was added before use. The protein concentration was determined using a Peirce BCA Protein Assay (Thermo Fisher Scientific). Cell lysates were separated on 10% sodium dodecyl sulfate polyacrylamide gel electrophoresis (SDS-PAGE) and were then transferred to polyvinylidene difluoride (PVDF) membranes (Millipore, Bedford, MA, USA). The membranes were blocked in Bullet Blocking One (Nacalai Tesque) for 5 min and were incubated with the primary antibodies followed by incubation with horseradish peroxidase (HRP)-conjugated secondary antibody (GE Healthcare, Buckinghamshire, UK). The bound antibodies were visualized using Chemi-Lumi One Ultra (Nacalai Tesque).

### 4.7. Small Interfering RNA Transfection

The knocking down the expression of OCT1 and *NCAPH* was conducted with small interfering RNA (siRNA) (10 nM) by a reverse transfection method using Lipofectamine RNAiMAX (Invitrogen, St. Louis, MO, USA) 48 h before harvesting the cells according to the manufacture’s protocols, unless otherwise indicated. Two specific siRNAs targeting OCT1 (siOCT1 #1 and siOCT1 #2), two specific siRNAs targeting *NCAPH* (si*NCAPH* #1 and si*NCAPH* #2), and one negative control siRNA targeting firefly luciferase (siControl) were purchased from Sigma-Aldrich. The sequences of the siRNAs were as follows: siOCT1 #1: 5′-GUGAAGGCUAGGUGAGUAAGC-3′; siOCT1 #2: 5′-GUGCUAGAUAGGUUUAUAAGU-3′; si*NCAPH* #1: 5′-UUUUUGAGCAUUCUAUAUACA-3′; si*NCAPH* #2: AAAUAACAGAUCAAUUUAGGA; siControl: 5′-GUGGAUUUCGAGUCGUCUUAA-3′.

### 4.8. Plasmid Construction and Transfection

We used the same OCT1 cDNA that we have used previously [8]. In brief, OCT1 cDNA was subcloned into mammalian expression plasmid pcDNA3.0 (Invitrogen), which was modified to generate the N-terminally flag-tagged protein. Expression vector encoding *NCAPH* was generated from the cDNA clone purchased from Promega (Madison, WI, USA). C-terminally flag-tagged *NCAPH* cDNA was amplified by polymerase chain reaction (PCR) with the following specific primers: *NCAPH* forward: 5′- CGGGATCCGCCACCATGGGACCTCCCGGCCCAG-3′; NCAPH-reverse: 5′- GCTCTAGATCACTTGTCATCGTCGTCCTTGTAGTCATCTCCTTGCCTCACAAGAACATC-3′. The generated amplicon was then subcloned into pcDNA3. The transfection of the expression vectors containing flag-tagged OCT1 or NACPH cDNA and empty vector alone was performed after 24 h using FuGENE HD (Promega), according to the manufacturer’s protocols. To establish stable transfectants, MCF-7 clones were selected using G418 (Nacalai Tesque) at a concentration of 600 μg/mL.

### 4.9. Cell Proliferation Assay

The cells were seeded at concentrations of 3.0 × 10^4^ cells/well (MCF-7) or 5.0 × 10^4^ cells/well (LTED), with indicated the siRNAs in a reverse transfection method in 96-well plates. Two kinds of clones stably expressing OCT1 (OCT1-OE #1 and #2) and cells transfected with empty vector (Vector) were seeded at the concentration of 2.0 × 10^4^ cells/well in 96-well plates. Cells were harvested on the 1st and 4th days, lysed with freeze-and-thaw, and buffered with 10 mM Tris-HCl, pH7.4, containing 100 mM NaCl and 1 mM EDTA. Extracted DNA was stained with Hoechst 33342 (Dojindo, Tokyo, Japan) at a final concentration of 20 μg/mL. The measurement of the DNA content in each well was performed using VICTOR Nivo (Perkin Elmer, Waltham, MA, USA). The wavelength of excitation was 355 nm, and the emission wavelength was 460 nm. Cell viability was evaluated by means of MTS assay after culturing the cells for the indicated duration using The Cell Titer 96 Aqueous One Solution Cell Proliferation Assay (Promega) according to the manufacturer’s instructions.

### 4.10. Flow Cytometric Analysis

The cells were harvested and fixed with 70% ethanol at −30 °C overnight. Fixed cells were treated with RNase A (Takara) and were stained with 50 μg/mL propidium iodide (PI) (Sigma-Aldrich). The cells were analyzed by means of a BD LSRFortessa (Becton Dickinson, Franklin Lakes, NJ, USA). The proportion of cells in the G0/G1, S, and G2/M phases of the cell cycle was evaluated using FlowJo software (Becton Dickinson).

### 4.11. Microarray Analysis

Total RNAs from MCF-7 cells transfected by a reverse transfection method with siRNAs (siControl, siOCT1 #1 and siOCT1 #2) were extracted by using Sepasol-RNA I super G (Nacalai Tesque) 48 h after the transfection of the siRNAs. For the gene expression microarray, the Clariom S Assay, human (Thermo Fisher Scientific) was used according to the manufacturer’s protocols. Data analysis was performed using Transcriptome Analysis Console (TAC) 4.0 Software. Data were deposited at Gene Expression Omnibus [49] as GEO accession number GSE179241.

### 4.12. ChIP Assay

ChIP and qRT-PCR were performed as previously described [10]. Briefly, for immunoprecipitation by the anti-OCT1 antibody, chromatin from crosslinked MCF-7 cells was sonicated and incubated with anti-OCT1 antibody or normal rabbit IgG at 4 °C overnight. The mixture was then incubated with protein G-Sepharose beads (GE Healthcare) at 4 °C for 2 h and were washed with radio-immunoprecipitation assay (RIPA) buffer, lithium buffer, and tris-EDTA (TE) buffer. The cross-linked DNA-protein complex was reversed through incubation at 65 °C overnight. Immunoprecipitated DNA was ethanol precipitated. The fold enrichment relative to the IgG control was measured by performing qPCR. The primer sequences for quantifying rgw OCT1-binding regions were as follows:

NCAPH promoter forward: 5′-CCCAAGAAGCCCAATCAGAC-3′;

NCAPH promoter reverse: 5′-CTTTCCTTGGCGTCTCCTG-3′;

Negative control forward: 5′-ACTGGTTAATGTGGTGACTGG-3′;

Negative control reverse: 5′-GGGTGAGGTATGGGCTAGAG-3′.

### 4.13. Statistical Analysis

SPSS Statics version 25 (IBM, Armonk, NY, USA) was used for all statistical analyses. Chi-square tests were used to evaluate the relationship between the immunoreactivity of the target protein and clinicopathological parameters. Disease-free survival was assessed using Kaplan–Meier curves, and statistical significance was calculated using the log-rank test. Univariate and multivariate analyses were evaluated using the Cox proportional hazard model. The statistical analyses of the qRT-PCR and proliferation assay were performed using Student’s t-test or one-way ANOVA followed by Dunnett’s test as a post hoc analysis. The *p*-value was based on the two-sided statistical analysis, and a value of *p* < 0.05 was considered to indicate a statistically significant difference.

## Figures and Tables

**Table 1:** Relationship between OCT1 immunoreactivity and clinicopathological parameters in ER-positive breast cancer patients.

		OCT1 Status		*p*-Value
		Positive IR (*n* = 48)	Negative IR (*n* = 60)	
**Age**	≤50 years old	26	27	0.344
	>50 years old	22	33	
**Stage**	I	17	25	0.508
	II, III	31	35	
**Invasive tumor size**	≤20 mm	26	34	0.795
	>20 mm	22	26	
**Lymph node status**	Positive	21	22	0.455
	Negative	27	38	
**Nuclear grade**	1	24	36	0.299
	2, 3	24	24	
**PgR status**	Positive	40	52	0.628
	Negative	8	8	
**HER2 status**	Positive	6	8	0.898
	Negative	42	52	

Abbreviations: ER, estrogen receptor; HER2, human epidermal growth factor receptor 2; IR, immunoreactivity; PgR, progesterone receptor.

**Table 2:** Univariate and multivariate analyses of disease-free survival with clinicopathological parameters including OCT1 immunoreactivity in ER-positive breast cancer patients.

Variables	Univariate	Multivariate		
	*p*-Value	Hazard Ratio	95% CI	*p*-Value
**Age** (≤50 vs. >50 years old)	0.186			
**Invasivetumor size** (≤20 vs. >20 mm)	**0.012**	2.39	0.99–5.75	0.052
**Lymph node status** (+ vs. −)	**0.014**	2.09	0.89–4.91	0.091
**Nuclear grade** (1 vs. 2, 3)	**0.007**	2.21	0.87–5.62	0.095
**PgR status** (+ vs. −)	0.628			
**HER2 status** (+ vs. −)	0.656			
**OCT1 IR** (+ vs. −)	**0.024**	2.38	1.03–5.52	**0.043**

Note: Significant *p*-values are expressed in bold. Abbreviations: CI, confidence interval; ER, estrogen receptor; HER2, human epidermal growth factor receptor 2; IR, immunoreactivity; PgR, progesterone receptor.

**Table 3:** Relationship between *NCAPH* immunoreactivity and clinicopathological parameters in ER-positive breast cancer patients.

		*NCAPH* Status		*p*-Value
		Positive IR (*n* = 63)	Negative IR (*n* = 45)	
**Age**	≤50 years old	32	21	0.344
	>50 years old	31	24	
**Stage**	I	21	21	0.161
	II, III	42	24	
**Invasive tumor size**	≤20 mm	35	25	1.000
	>20 mm	28	20	
**Lymph node status**	Positive	28	15	0.245
	Negative	35	30	
**Nuclear grade**	1	31	29	0.116
	2, 3	32	16	
**PgR status**	Positive	51	41	0.143
	Negative	12	4	
**HER2 status**	Positive	9	5	0.628
	Negative	54	40	
**OCT1 status**	Positive IR	42	6	**<0.001**
	Negative IR	21	39	

Note: Significant *p*-value is expressed in bold. Abbreviations: ER, estrogen receptor; HER2, human epidermal growth factor receptor 2; IR, immunoreactivity; PgR, progesterone receptor.

**Table 4:** Univariate and multivariate analyses of disease-free survival with clinicopathological parameters including *NCAPH* immunoreactivity in ER-positive breast cancer patients.

Variables	Univariate	Multivariate		
	*p*-Value	Hazard Ratio	95% CI	*p*-Value
**Age** (≤50 vs. >50 years old)	0.186			
**Invasive tumor size** (≤20 vs. >20 mm)	**0.012**	2.76	1.15–6.67	**0.024**
**Lymph node status** (+ vs. −)	**0.014**	2.22	0.95–5.19	0.066
**Nuclear grade** (1 vs. 2, 3)	**0.007**	2.15	0.85–5.44	0.105
**PgR status** (+ vs. −)	0.628			
**HER2 status** (+ vs. −)	0.656			
***NCAPH* IR** (+ vs. −)	**0.049**	2.61	1.01–6.78	**0.048**

Note: Significant *p*-values are expressed in bold. Abbreviations: CI, confidence interval; ER, estrogen receptor; HER2, human epidermal growth factor receptor 2; IR, immunoreactivity; PgR, progesterone receptor.

## Data Availability

The microarray data were deposited at Gene Expression Omnibus [49] as GEO accession number GSE179241.

## Supplementary Materials

The following are available online at https://www.mdpi.com/article/10.3390/ijms222111505/s1, Table S1: Sixteen genes commonly regulated by siOCT1 #1 and siOCT1 #2 in MCF-7 cells, Figure S1: Specificity of anti-OCT1 antibody, Figure S2: Analysis of OCT1 expression using public database, Figure S3: OCT1 expression in breast cancer cells, Figure S4: OCT1 promotes cell proliferation and induces the cell cycle transition from G1 phase to S phase, Figure S5: Kaplan–Meier survival analysis of breast cancer patients for candidate OCT1-target genes identified in the present study, Figure S6: Specificity of anti-*NCAPH* antibody, Figure S7: *NCAPH* expression in breast cancer cells, Figure S8: Knockdown of *NCAPH* inhibited the cell cycle transition from G1 phase to S phase, Figure S9: Overexpression of *NCAPH* rescued the suppressed cell cycle transition from G1 phase to S phase by siOCT1.
