# Supplementary material for: OCT1 Is a Poor Prognostic Factor for Breast Cancer Patients and Promotes Cell Proliferation via Inducing NCAPH
Source: Int J Mol Sci. 2021 Oct 25;22(21):11505. doi: 10.3390/ijms222111505 (PMC8584020; doi:10.3390/ijms222111505)
Supplement: Supplementary file 1 [file ijms-22-11505-s001.zip › ijms-1415532-supplementary.pdf]

Supplementary Material

OCT1 is a poor prognostic factor for breast cancer patients and promotes cell proliferation via inducing NCAPH

Takuya Ogura [1,2,3], Kotaro Azuma [1], Junichiro Sato [4], Keiichi Kinowaki [4], Ken-Ichi Takayama [1], Toshihiko Takeiwa [1], Hidetaka Kawabata [2], Satoshi Inoue [1,5,*]

1 Department of Systems Aging Science and Medicine, Tokyo Metropolitan Institute of Gerontology, 35-2 Sakae-cho, Itabashi-ku, Tokyo 173-0015, Japan; ogutaku0120@gmail.com (T.O.); azumak@m.u-tokyo.ac.jp (K.A.); takayama@tmig.or.jp (K.T.); ttakeiwa@tmig.or.jp (T.T.)

2 Department of Breast and Endocrine Surgery, Toranomon Hospital, 2-2-2 Toranomon, Minato-ku, Tokyo 105-8470, Japan; h-kawabata@toranomon.gr.jp (H.K.)

3 Department of Systems BioMedicine, Tokyo Medical and Dental University, 1-5-45 Yushima, Bunkyo-ku, Tokyo 113-8510, Japan

4 Department of Pathology, Toranomon Hospital, 2-2-2 Toranomon, Minato-ku, Tokyo 105-8470, Japan; j.n.sato@nifty.com (J.S.); kinowaki-hok@umin.ac.jp (K.K.)

5 Division of Systems Medicine and Gene Therapy, Saitama medical University, 1397-1 Yamane, Hidaka-shi, Saitama 350-1241, Japan

* Correspondence: sinoue@tmig.or.jp; Tel.: +81-3-3964-3241

Table of contents

Table S1	Page 2
Figure S1	Page 3
Figure S2	Page 4
Figure S3	Page 5
Figure S4	Page 6
Figure S5	Page 7
Figure S6	Page 8
Figure S7	Page 9
Figure S8	Page 10
Figure S9	Page 11

Table S1 Sixteen genes commonly regulated by siOCT1 #1 and siOCT1 #2 in MCF-7 cells

Gene symbol	Description	siCont. vs siOCT1 #1 Fold change	siCont. vs siOCT1 #2 Fold change
HIST1H1B	histone cluster 1, H1b	14.6	26.9
RRM2	ribonucleotide reductase M2	23.4	23.9
HIST1H2BM	histone cluster 1, H2bm	13.9	27.5
HIST2H3C	histone cluster 2, H3c	9.8	47.0
HIST2H3A	histone cluster 2, H3a	9.1	41.1
MYB	v-myb avian myeloblastosis viral oncogene homolog	29.7	14.1
CDCA3	cell division cycle associated 3	9.4	20.0
MGP	matrix Gla protein	10.3	15.1
DTL	denticleless E3 ubiquitin protein ligase homolog (Drosophila)	14.1	10.2
NCAPH	non-SMC condensin I complex subunit H	9.4	11.0
PBK	PDZ binding kinase	10.3	10.2
CEP55	centrosomal protein 55kDa	15.1	9.2
TMEM64	transmembrane protein 64	66.0	8.6
HIST1H1D	histone cluster 1, H1d	8.5	21.8
KIF20A	kinesin family member 20A	8.3	26.5
HIST1H3I	histone cluster 1, H3i	8.2	19.4

Genes downregulated by 8 fold change or more in both treatments with siOCT1 #1 and siOCT1 #2 are shown. Ratios of expression levels (expression level with siControl treatment divided by that with siOct1 treatment) are displayed as fold changes. Abbreviation: siCont., siControl.

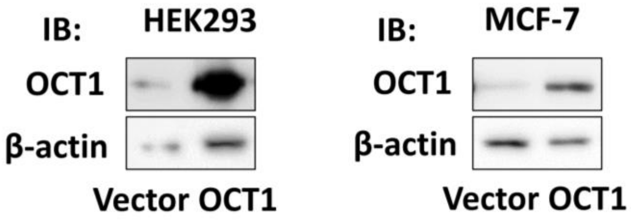

Figure S1 Specificity of anti-OCT1 antibody. HEK293 cells and MCF-7 cells were transiently transfected with expression vector encoding OCT1 (OCT1). Empty vector (Vector) was used as a negative control. Cell lysates were separated by sodium dodecyl sulfate polyacrylamide gel electrophoresis (SDS-PAGE), and immunoblot analysis was performed. An antibody against β-actin was used as a loading control. IB, immunoblot.

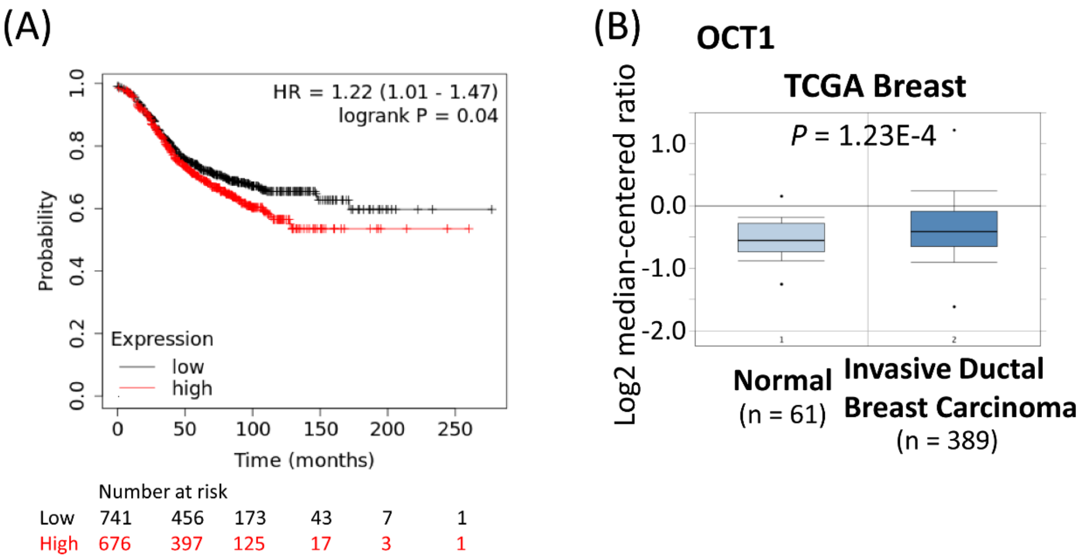

Figure S2 Analysis of *OCT1* expression using public database. A, Kaplan-Meier survival analysis of the association of recurrence-free survival rate with *OCT1* expression in ER-positive breast cancer patients using Kaplan-Meier Plotter database. Available online: http://www.kmplot.com (accessed 25th October 2021). Auto-selected best cutoff was employed to split the patients. B, *OCT1* expression levels in invasive ductal breast carcinoma tissues (*n* = 61) was compared with that in normal breast tissues (*n* = 389) using a public database (TCGA Breast) in Oncomine. Available online: https://www.oncomine.org/resource/login.html (accessed 25th October 2021).

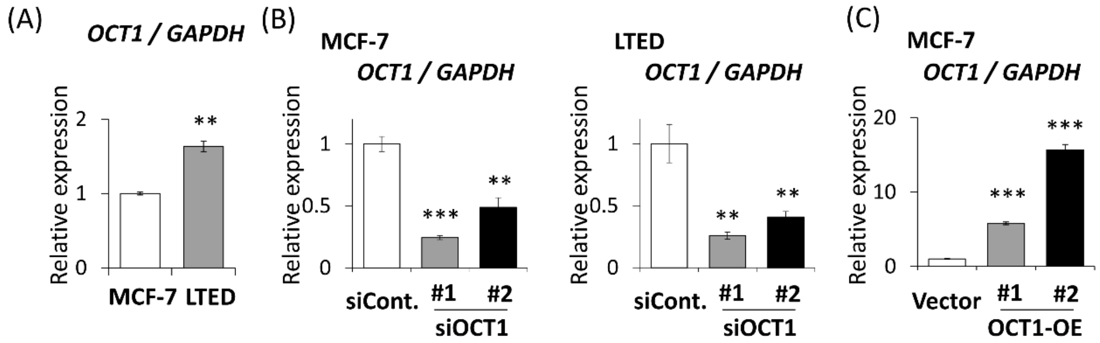

Figure S3 *OCT1* expression in breast cancer cells. A, Expression of *OCT1* in MCF-7 cells and LTED cells was analyzed by qRT-PCR. The relative mRNA levels were normalized with *GAPDH* expression and presented as mean and SEM (n = 3). $^{**}P < 0.01$. B, Expression of *OCT1* in MCF-7 cells and LTED cells treated with two kinds of siRNAs for OCT1 (siOCT1 #1 or #2) or siControl (siCont.) was analyzed by qRT-PCR. The relative mRNA levels were normalized with *GAPDH* expression and presented as mean and SEM (n = 3). $^{**}P < 0.01$, $^{***}P < 0.001$, compared with cells treated with siControl. C, Expression of *OCT1* in two clones of MCF-7 cells stably expressing OCT1 (OCT1-OE #1 and #2) and an MCF-7 clone transfected with empty vector (Vector) was analyzed by qRT-PCR. The relative mRNA levels were normalized with *GAPDH* expression and presented as mean and SEM (n = 3). $^{***}P < 0.001$, compared with Vector.

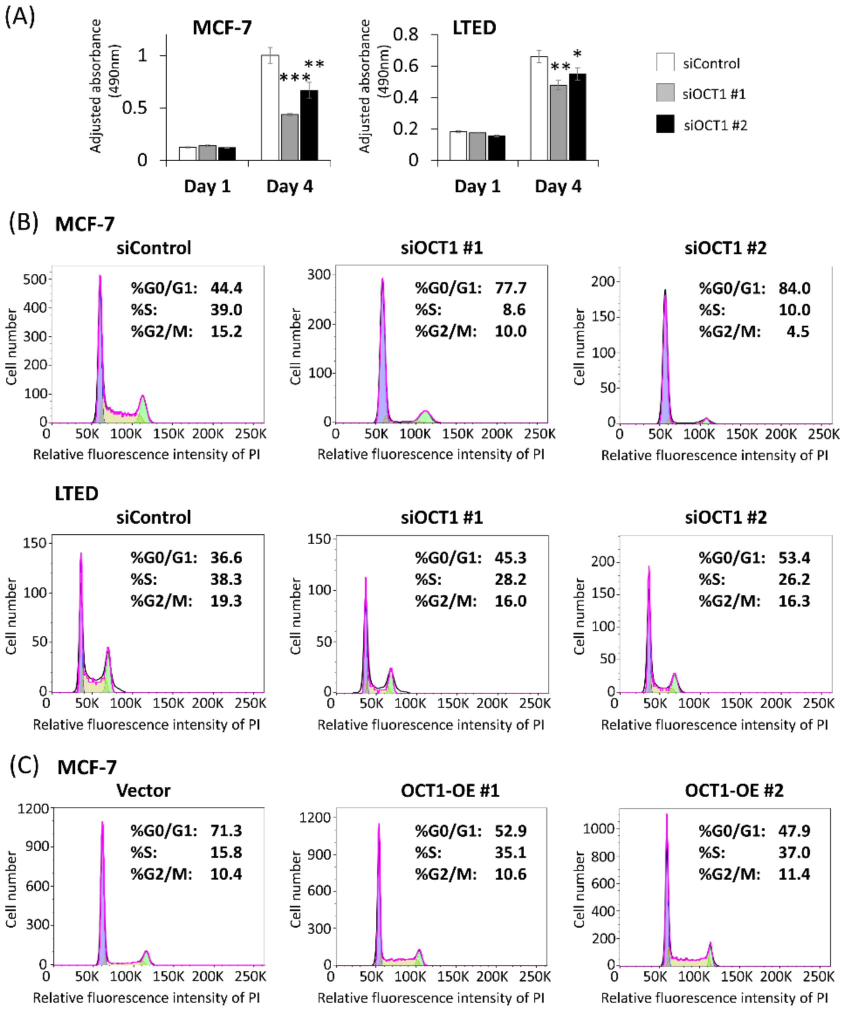

Figure S4 OCT1 promotes cell proliferation and induces the cell cycle transition from G1 phase to S phase. A, Inhibitory effect by siRNAs for OCT1 on the proliferation of MCF-7 and LTED cells. MTS assay was performed on indicated days after transfection of two kinds of siRNAs for OCT1 (siOCT1 #1 or #2) or siControl. Results are expressed as mean and SEM (n = 4). **P* < 0.05, ***P* < 0.01, ****P* < 0.001, compared with cells treated with siControl. B, Propidium iodide (PI) histogram plots obtained by flow cytometric analysis of MCF-7 and LTED cells transfected with indicated siRNAs. The proportion of cells in the G0/G1 phase (blue area), S phase (yellow area), and G2/M phase (green area) of the cell cycle was determined by FlowJo software. C, PI histogram plots obtained by flow cytometric analysis of MCF-7 clones stably expressing OCT1 (OCT1-OE #1 and #2) and an MCF-7 clone transfected with empty vector (Vector). The proportion of cells in the G0/G1 phase (blue area), S phase (yellow area), and G2/M phase (green area) of the cell cycle was determined by FlowJo software.

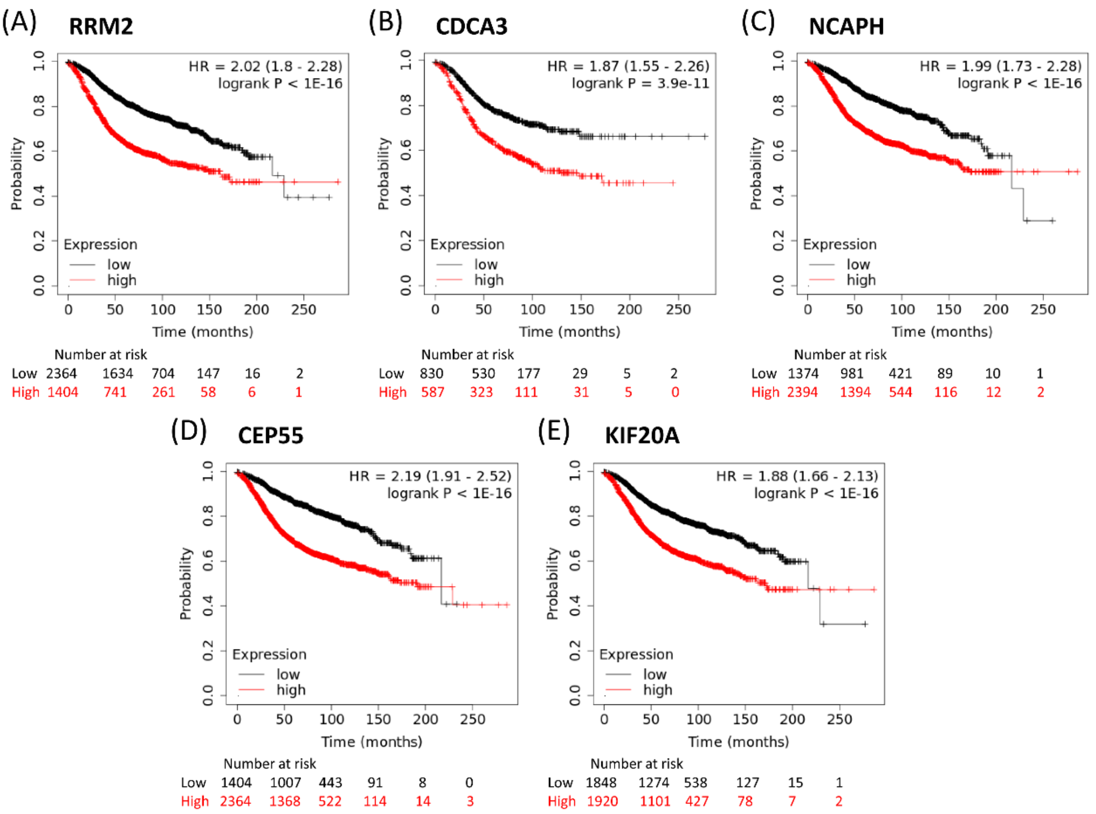

Figure S5 Kaplan-Meier survival analysis of breast cancer patients for candidate OCT1-target genes identified in the present study. A-E, Analyses of the association of recurrence-free survival rate with expression of indicated genes in ER-positive breast cancer using Kaplan-Meier Plotter database. Availabe online: http://www.kmplot.com (accessed 25th October 2021). Auto-selected best cutoff was employed to split the patients.

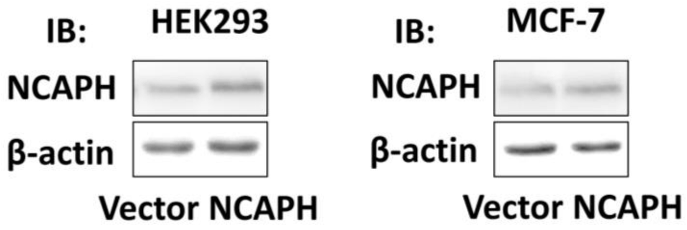

Figure S6 Specificity of anti-NCAPH antibody. HEK293 cells and MCF-7 cells were transiently transfected with expression vector encoding NCAPH (NCAPH). Empty vector (Vector) was used as a negative control. Cell lysate were separated by SDS-PAGE, and immunoblot analysis was performed. An antibody against β-actin was used as a loading control.

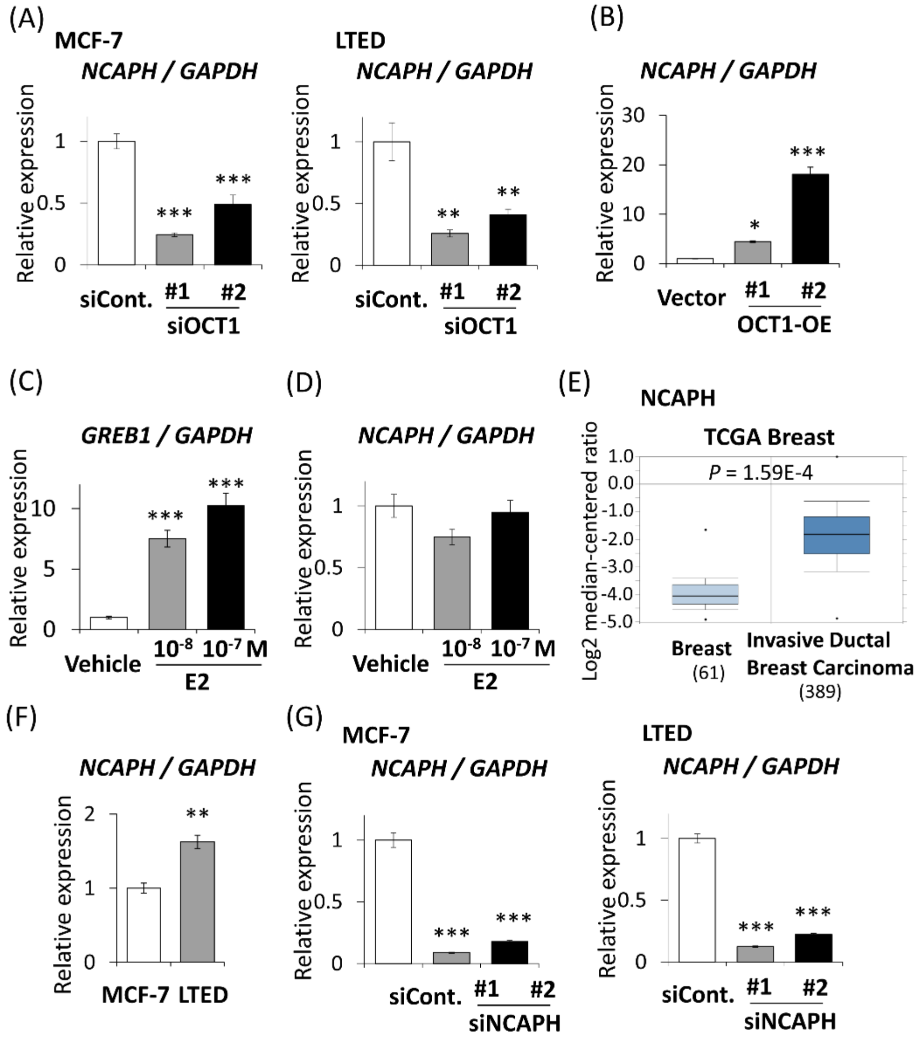

Figure S7 *NCAPH* expression in breast cancer cells. A, Expression of *NCAPH* in MCF-7 cells and LTED cells treated with two kinds of siRNAs for OCT1 (siOCT1 #1 or #2) or siControl (siCont.) was analyzed by qRT-PCR. The relative mRNA levels were normalized with *GAPDH* expression and presented as mean and SEM (n = 3). **$P < 0.01$, ***$P < 0.001$, compared with cells treated with siControl. B, Expression of *NCAPH* in two clones of MCF-7 cells stably expressing OCT1 (OCT1-OE #1 and #2) and an MCF-7 clone transfected with empty vector (Vector) was analyzed by qRT-PCR. The relative mRNA levels were normalized with *GAPDH* expression and presented as mean and SEM (n = 3). *$P < 0.05$, *$P < 0.001$, compared with Vector. C, MCF-7 cells were treated with the indicated concentrations of 17β-estradiol (E2) or 0.1% ethanol (Vehicle) for 24 h. *GREB1* expression were analyzed by qRT-PCR. The relative mRNA levels were normalized with *GAPDH* expression and presented as mean and SEM (n = 3). ***$P < 0.001$, compared with cells treated with ethanol. D, *NCAPH* expression in the same sample as in Figure S5C was analyzed by qRT-PCR. The relative mRNA levels were normalized with *GAPDH* expression and presented as mean and SEM (n = 3). E, *NCAPH* mRNA expression levels in invasive ductal breast carcinoma tissues (n = 61) was compared with that in normal breast tissue (n = 389) using a public database (TCGA Breast) in Oncomine. Avilable online: https://www.oncomine.org/resource/login.html (accessed 25th October 2021). F, Expression of *NCAPH* in MCF-7 cells and LTED cells was analyzed by qRT-PCR. The relative mRNA levels were normalized with *GAPDH* expression and presented as mean and SEM (n = 3). **$P < 0.01$. G, Expression of *NCAPH* in MCF-7 cells and LTED cells treated with two kinds of siRNAs for NCAPH (siNCAPH1 #1 or #2) or siControl (siCont.) was analyzed by qRT-PCR. The relative mRNA levels were normalized with *GAPDH* expression and presented as mean and SEM (n = 3). ***$P < 0.001$, compared with cells treated with siControl.

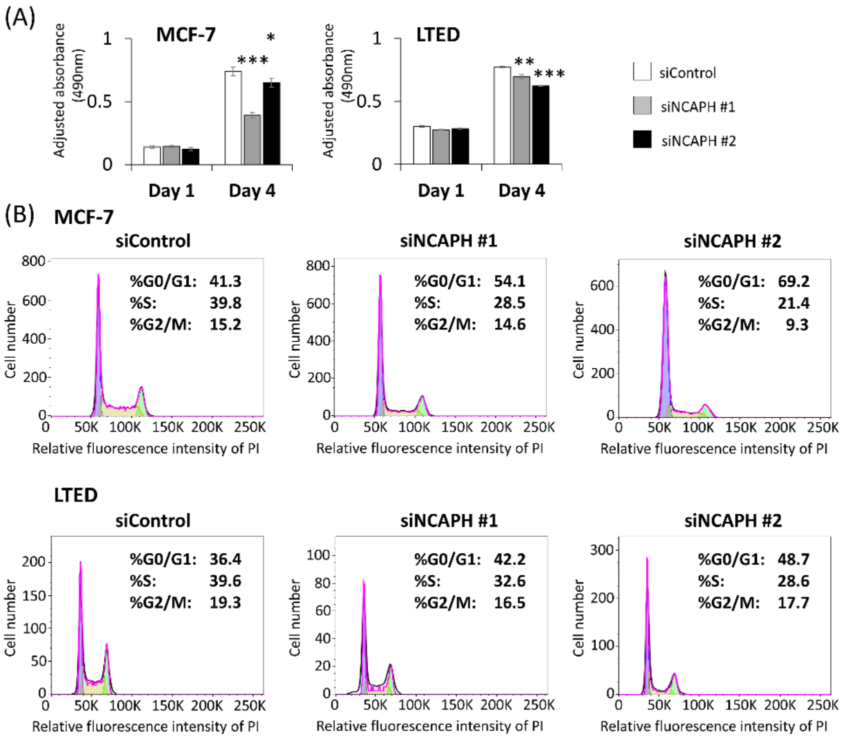

Figure S8. Knockdown of NCAPH inhibited the cell cycle transition from G1 phase to S phase. A, Inhibitory effect by siRNAs for NCAPH on the proliferation of MCF-7 and LTED cells. MTS assay was performed on indicated days after transfection of two kinds of siRNAs for NCAPH (siNCAPH #1 or #2) or siControl. Results are expressed as mean and SEM (n = 4). *$P < 0.05$, **$P < 0.01$, ***$P < 0.001$, compared with cells treated with siControl. B, Propidium iodide (PI) histogram plots obtained by flow cytometric analysis of MCF-7 and LTED cells transfected with indicated siRNAs. The proportion of cells in the G0/G1 phase (blue area), S phase (yellow area), and G2/M phase (green area) of the cell cycle was determined by FlowJo software.

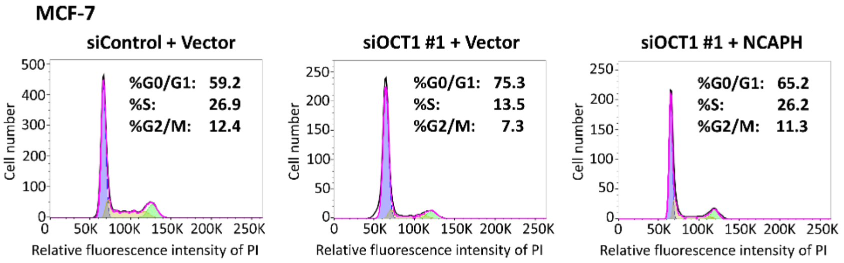

Figure S9. Overexpression of NCAPH rescued the suppressed cell cycle transition from G1 phase to S phase by siOCT1. Propidium iodide (PI) histogram plots obtained by flow cytometric analysis of MCF-7 cells transfected with indicated siRNAs were shown. On the next day of transfection with siRNAs (siControl or siOCT1 #1), transfection with expression vector encoding NCAPH (NCAPH) or empty vector (Vector) was performed. The cells were fixed two days after transfection with the vector encoding NCAPH or the empty vector and subjected to the flow cytometric analysis. The proportion of cells in the G0/G1 phase (blue area), S phase (yellow area), and G2/M phase (green area) of the cell cycle was determined by FlowJo software.
